# Real-time virtual sonography-assisted radiofrequency ablation in liver tumors with conspicuous or inconspicuous images or peritumoral landmarks under ultrasonography

**DOI:** 10.1007/s00261-020-02875-y

**Published:** 2021-01-02

**Authors:** Hsin-Chih Huang, L. B. Gatchalian, Yi-Chung Hsieh, Wei-Ting Chen, Chen-Chun Lin, Shi-Ming Lin

**Affiliations:** 1 Department of Gastroenterology and Hepatology, Linkou Chang Gung Memorial Hospital, Taoyuan, Taiwan; 2 Liver Research Unit, Chang Gung Memorial Hospital, Chang Gung University, 199, Tung Hwa North Road, Taipei, Taiwan

**Keywords:** Real-time virtual sonography, Radiofrequency ablation, Hepatocellular carcinoma, Liver metastasis, Inconspicuous tumor

## Abstract

**Objectives:**

The objectives of this study were to determine the primary technique effectiveness (PTE), to compare the complete response and local recurrence rates between conspicuous and inconspicuous tumors using single and switching electrodes of real-time virtual sonography (RVS)-assisted radiofrequency ablation (RFA) in conspicuous and inconspicuous hepatic tumors under conventional ultrasonography (US).

**Subjects and method:**

We compared the complete ablation of inconspicuous tumors with and without anatomical landmark (*N* = 54) with conspicuous liver tumors (*N* = 272). Conventional US imaging was done initially, and then these images were fused with CT or MRI arterial-venous-wash-out cross-sectional studies and synchronized with real-time US images.

**Results:**

RVS-assisted RFA was technically feasible in all patients. The PTE rate after the first ablation was 94% (245/261) for conspicuous tumors, 88% (7/8) in inconspicuous tumors with landmark, and 78% (36/46) in inconspicuous tumors without landmark. The complete response (*p* = 0.1912 vs. *p* = 0.4776) and local recurrence rate (*p* = 0.1557 vs. *p* = 0.7982) were comparable in conspicuous tumors of both HCC and liver metastasis group when single or multiple switching was used. The cumulative local recurrence in the conspicuous and inconspicuous tumors of the HCC group (*p* = 0.9999) was almost parallel after 12 (10% vs. 4%) and 24 (13% vs. 4%) months of follow-up. In the liver metastasis group, the cumulative local recurrence for conspicuous tumors (*p* = 0.9564) was nearly equal after 12 and 24 months of monitoring (24% vs. 27%) while no recurrence was incurred for the inconspicuous tumors.

**Conclusion:**

RVS-assisted RFA is an effective tool for the treatment of conspicuous and inconspicuous HCC and hepatic metastasis.

## Introduction

Hepatocellular carcinoma (HCC) is the most common primary liver malignancy. The liver is also a frequent site for metastatic tumor spread. Numerous studies focus on the various therapeutic modalities for HCC treatment depending on tumor size and number, vascular invasion, presence of portal hypertension, comorbidities, and performance status of patients [1–5]. Optimal individualized care requires a multispecialty team. Radiofrequency ablation (RFA) is a widely recognized and effective locoregional treatment for primary HCC and liver metastasis. Its role for HCC can be both palliative and curative. However, its use for liver metastasis is for palliation [6] due to the advanced or unresectable tumor stage. Its mechanism is to eradicate focal tumors by coagulation necrosis [7].

It is a challenge for every interventional hepatologist or radiologist to appropriately identify the liver lesions. However, there are some lesions that are indistinct by conventional ultrasound, and the advent of real-time virtual sonography (RVS) has augmented in the precise identification of liver tumors during RFA making it efficient and safe. Most of the published studies use RVS to detect tumors and guide RFA in the treatment of inconspicuous primary HCC [8–14]. Some studies also showed the usefulness of RVS in inconspicuous hepatic metastasis [6, 10, 13, 14].

This study used RVS in both conspicuous and inconspicuous primary HCC and metastatic hepatic tumors. The primary objective of this study was to determine the primary technique effectiveness of RFA with RVS guidance in conspicuous and inconspicuous (with or without peritumoral anatomical landmark) hepatic tumors. The secondary objectives were to compare the complete response and local recurrence rates between conspicuous and inconspicuous tumors using single and multiple electrodes with switch, and to assess the cumulative local recurrence in HCC and liver metastasis groups.

## Materials and methods

This is a retrospective study from January 2015 to December 2016 which included patients with liver tumors (HCC or metastasis) who underwent first-time RFA. All tumors were diagnosed using computed tomography or magnetic resonance contrast imaging, cytology, and pathology. The patient demographics and tumor characteristics were identified (Table 1).

**Table 1:** Demographics of patients and tumors

Variables	HCC group	Liver metastasis group	*p* value
Number of patients	196	23	–
Male	127	18	0.173
Female	69	5	
Age (y.o.)			
Mean + / − SD	65.2 + / − 11.3	59 + / − 15.6	*0.008*
Range	10 – 90	7 – 83	
Tumor Size(cm) + / − SD(range)	2.18 + / − 0.8725	2.24 + / − 0.9595	0.6778
Complete response of tumor (CT or MRI)	249/272 (92%)	39/43 (91%)	0.1462

*p* value of < 0.10 was statistically significant

### Study design (Fig. 1)

**Fig. 1:**
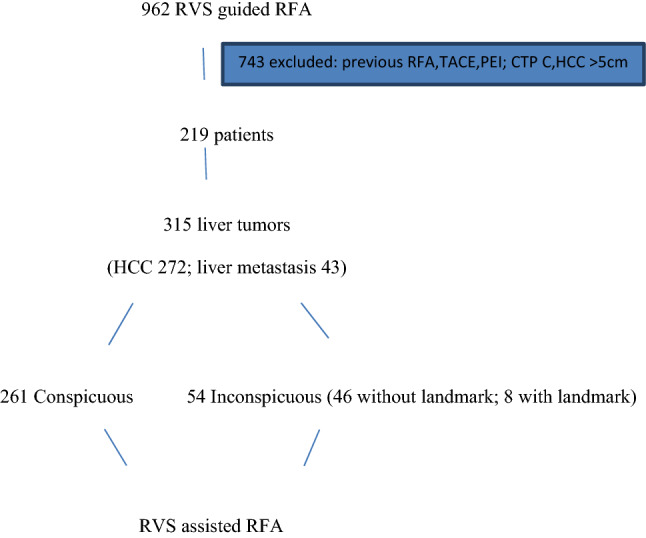
Flow diagram

**Fig. 2:**
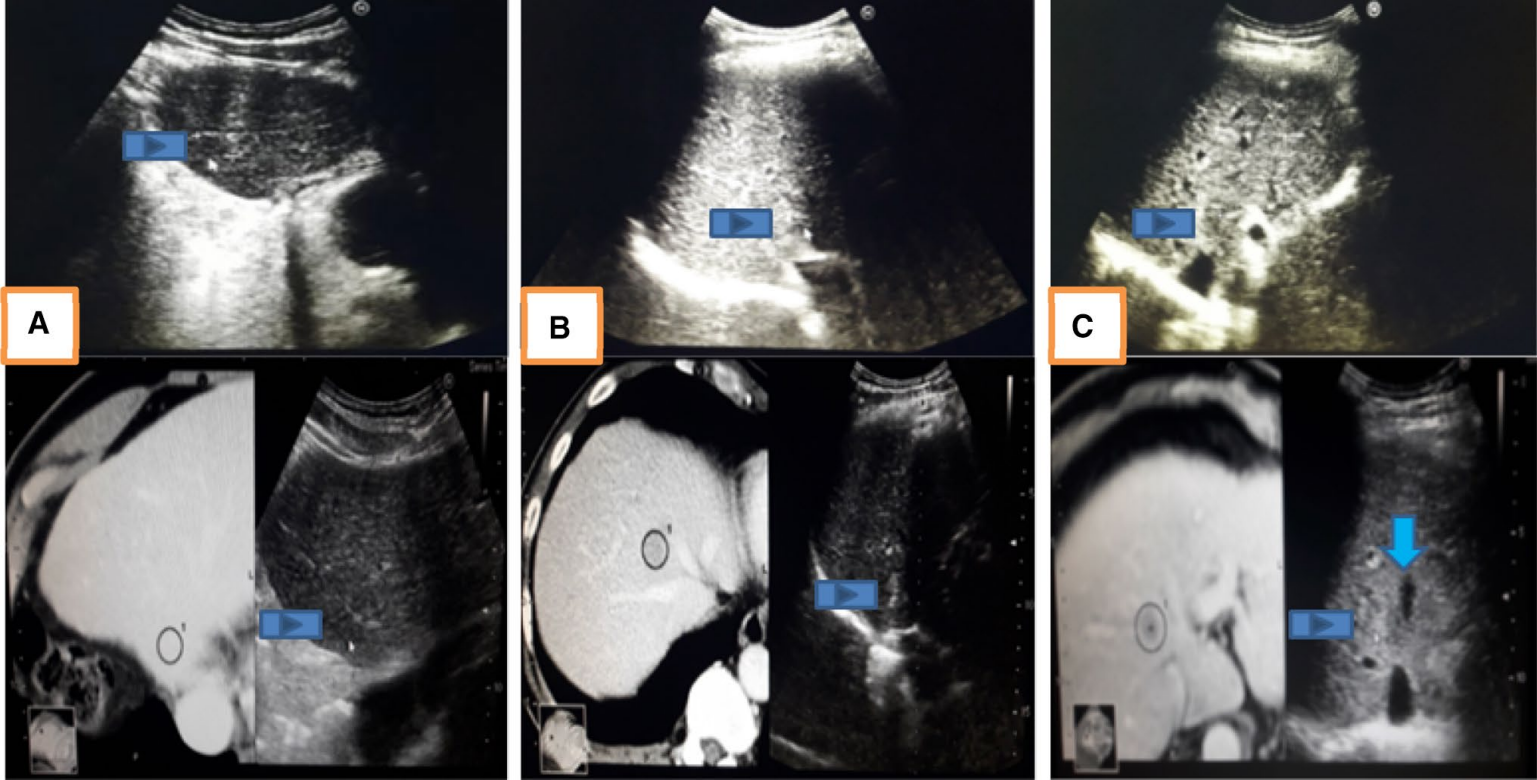
The images above show the tumor visibility under ultrasound and under RVS. **a** Conspicuous S4 tumor with visible margin and echogenicity **b** Inconspicuous S8 tumor without visible margin **c** Inconspicuous S8 tumor without visible margin, beside middle hepatic vein

A total of 962 patients underwent RVS-assisted RFA in our liver unit from January 2015 to December 2016. Seven hundred forty-three (743) patients were excluded due to their previous treatments (739 patients with previous RFA, TACE, PEI), CTP C (2 patients), and > 5 cm tumor sizes (2 patients). Thus, a total of 219 patients with 315 liver tumors who underwent first-time RFA with the aid of RVS were included from the database of four interventional hepatologists. Conventional ultrasound using convex probe was done, and tumors were classified as conspicuous or inconspicuous (Fig. 2). Based on the study of CH Lee et al. [15], a detectable lesion under conventional US was defined as a lesion where the margin and echogenicity of the index tumor was visible, whereas an undetectable lesion was one in which the margin and echogenicity of the index tumor were not visible. In the inconspicuous tumor group, we subgrouped patients based on the presence or absence of peritumoral anatomical landmark (Fig. 2). A fixed anatomical landmark with less than 1 cm distance from index tumor was used since these landmarks (vessels and bile duct) were contributory factors in the identification of the tumors. A peritumoral anatomic landmark was defined as any ultrasound-discernible structure near the index tumor such as focal hepatic lesions (cysts, calcification, and previous ablation zone), bifurcation of the hepatic or portal vein, or liver configuration [16]. CT or MRI arterial-venous-wash-out cross-sectional studies were synchronized with real-time US images [17, 18].

### RFA protocol

A 17-cm gauge radiofrequency electrode with 2–3 cm active tip and an internal cooling system attached to the radiofrequency generators were used. In some cases, multiple RF electrodes with switch control were utilized. Either artificial ascites or pleural effusion was done prior to RFA for tumors in high-risk locations. After application of aseptic technique, 10 ml of 1% lidocaine was injected through the skin. The RF electrodes were inserted percutaneously into the target tumor under real-time US with RVS guidance. All RFAs were done under conscious sedation with the use of Midazolam and Fentanyl. The vital signs and oxygen saturation were monitored during the procedure. Patients were discharged less than 48 hours after post-RFA ultrasound evaluation.

### Follow-up protocol

Contrast imaging with CT or MRI was done within 1 month after RFA to assess complete ablation or residual viable tumors in both HCC and metastasis group. A complete response was confirmed by a dynamic CT showing a widely low-density area with sufficient safety margin [9]. A tumor was labeled as residual and viable if it was with peripheral enhancement in the arterial phase [12]. Primary technique effectiveness is defined as prospectively defined time point at which complete ablation of macroscopic tumor, as evidenced by imaging follow-up [7]. In this study, we classified as primary technique effectiveness if first follow-up contrast CT or MRI after RFA did not show any viable tumor and with sufficient safety margin. Repeat RFA, booster PEI, and TACE were options for viable tumors based on the discretion of the attending physicians. For tumors that underwent second and third RFA sessions, complete response to ablation was also assessed by contrast imaging. Contrast CT or MRI was then repeated every 3 months to assess local recurrence in tumors with complete response after first ablation. Local recurrence was defined as new tumor focus at the ablative margin after local eradication of all tumor cells with ablation [7].

### Statistical analysis

All tumors were assessed for complete ablation after each RFA session. Descriptive statistics were expressed as mean + / − SD. Logistic regression analysis was used to determine correlation of factors and outcome. The cumulative LR was analyzed using a Kaplan–Meier model. Statistical analysis was conducted using SPSS version 20 software. A *p* value < 0.10 was considered statistically significant.

## Results

The study population included 196 patients in the primary HCC group and 23 patients in the liver metastasis group. The HCC group had older patients with a mean age of 65.2 (Table 1). There were a total of 315 liver tumors (272 Primary HCC, 43 liver metastasis) identified. The mean diameter of the tumors was comparable at 2.18 cm + / − 0.8725 in the HCC group and 2.24 cm + / − 0.9595 in the liver metastasis group. Complete response to ablation after the first RFA was comparable between the 2 tumor groups (*p* = *0.1462*) (Table 1).

**Fig. 3:**
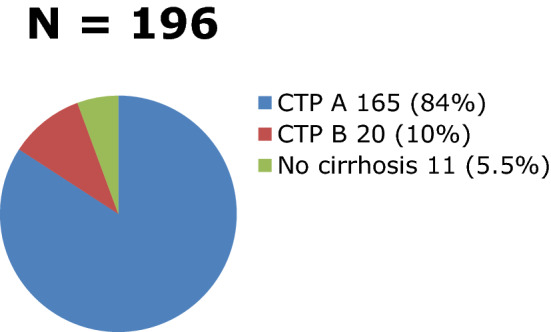
Distribution of Cirrhotic and Non-cirrhotic Patients

**Fig. 4:**
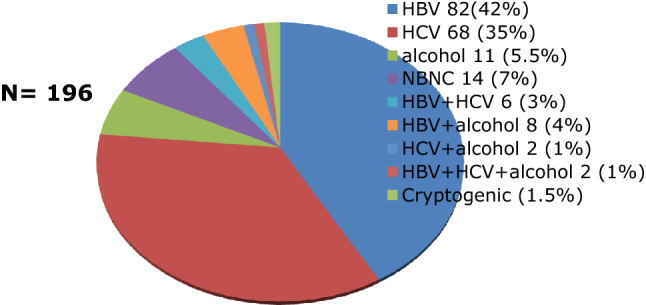
Etiologies of liver disease in HCC group

**Fig. 5:**
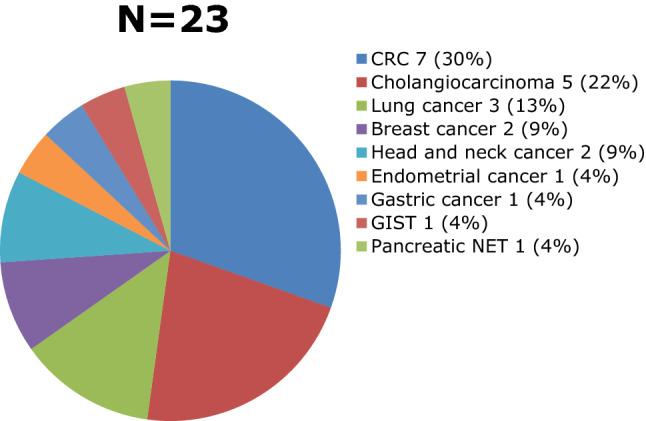
Distribution of Primary Malignancy with Hepatic Metastasis

Most of the 185 patients in the HCC group had liver cirrhosis under CTP A while 11 patients were non-cirrhotic (Fig. 3). The mean alpha-fetoprotein level in the HCC group was 189.3 ng/ml ± 1824.14. The well-known risk factors for liver disease identified were Hepatitis B, Hepatitis C, Non-B Non-C (NASH, Primary Biliary Cirrhosis), and alcohol intake. Ten percent (10%) of the HCC patients had more than one etiology (Fig. 4). Expectedly, half of the causes of liver metastasis were from the gastrointestinal tract (Fig. 5). All of these patients were safely treated with RFA (Table 2).

**Table 2:** Primary Technique Effectiveness of RVS-assisted RFA

	Conspicuous *N* = 261	Inconspicuous *N* = 54		*p* value
Size (cm) mean + / − SD	2.32 + / − 0.8618	1.54 + / − 0.6826		*0.000*
Primary technique effectiveness	245 (94%)	43 (80%)		0.9993
		With landmark *N* = 8	Without landmark *N* = 46	0.549
		7 (88%)	36 (78%)	

*p* value of < 0.10 was statistically significant

Conspicuous tumors had significantly larger size at 2.32 cm compared to inconspicuous tumors with a mean diameter of 1.54 cm *(p* = *0.000).* Furthermore, the sensitivity of tumor detection by B-mode US was 83% (261/ 315), and with the use of RVS, the sensitivity increased to 100% (261 + 54/ 315), thus, identifying the inconspicuous tumors. In the study of Okamoto [19] with 140 nodules in 59 patients, the sensitivity of conventional sonography was 50.71%, but with virtual CT sonography, the sensitivity increased to 83.57%.

The primary technique effectiveness of RFA with RVS guidance was higher in the conspicuous tumor group with a complete ablation rate in a single session at 94% (245/261); similarly, in the inconspicuous tumor group, it was at 80% (43/54) (*p* = *0.9993*)*.* In the inconspicuous tumor group without landmark, it was at 78% (36/46), while in the 8 tumors with landmark (vessels and bile duct), the complete ablation rate was at 88% (7/8). However, the relevance of these landmarks may not be statistically significant (*p* = *0.549*) due to the small sample size (*N* = 8) in the group (Table 2).

Table 3 shows that there were 261 conspicuous tumors that underwent first session of RFA, and 94% (245/261) achieved a complete response, while 6% (16/261) had a non-complete response. There were 8 tumors that underwent a 2nd RFA; however, only 75% (6/8) attained a complete response. The remaining 7 tumors did not undergo further RFA treatment, while 1 tumor was subjected to TACE. There were 2 tumors that were completely ablated during the 3rd RFA (100%).

Fifty-four inconspicuous tumors were divided into tumors with (*N* = 8) and without (*N* = 46) an anatomical landmark that underwent a first session of RFA. Seventy-eight percent (36/46) of inconspicuous tumors without landmark achieved a complete response, while 22% (10/46) had a non-complete response. Six tumors (6) had a 2nd RFA and attained a complete ablation (100%). Three tumors (3) were subjected to TACE, while the remaining tumor (1) did not undergo further treatment.

Eight inconspicuous tumors with landmark were treated with first-time RFA, and 88% (7/8) had a complete response while 12% (1/8) had a non-complete response. This tumor was located near a bile duct and subsequently underwent a second RFA however there was failure to achieve a complete response. No further RFA treatment was done.

Thus, RVS was a valuable tool in achieving a complete response during RFA (Table 3).

**Table 3:** Complete response per RFA session

RFA session	Complete response	Non-complete response
1st RFA *N* = 315		
Conspicuous *N* = 261	245 (94%)	16 (6%)
Inconspicuos *N* = 54		
With landmark *N* = 8	7 (88%)	1 (12%)
Without landmark *N* = 46	36 (78%)	10 (22%)
2nd RFA *N* = 15		
Conspicuous *N* = 8	6 (75%)	2 (25%)
Inconspicuous		
With landmark *N* = 1	0	1 (100%)
Without landmark *N* = 6	6 (100%)	0
3rd RFA *N* = 2		
Conspicuous *N* = 2	2(100%)	0
Inconspicuous *N* = 0	NA	NA

A total of 224 HCC tumors and 37 metastatic hepatic tumors underwent RFA using either single or multiple electrodes with switch. A complete response was achieved in 94% (191/204) of tumors in the HCC group that used a single electrode while 95% (19/20) in the switch type. The local recurrence rate was at 14% (27/191) and 16% (3/19), respectively, using the single and switch electrodes. By comparing the 2 groups, they were not statistically significant (*p* value > 0.1).

In the liver metastasis group, 100% (6/6) had a complete response with the switch electrode while only 94% (29/31) in the single electrode. The local recurrence rate was at 50% (3/6) and 24% (7/29) for the switch and single electrodes, respectively. The results were not statistically significant (*p* value > 0.1). (Table 4).

**Table 4:** Complete response and local recurrence rate in conspicuous tumors with use of single and multiple electrodes with switch

Electrode type	HCC *N* = 224				Liver metastasis *N* = 37			
	Complete response	*p* value	LR rate	*p* value	Complete response	*p* value	LR rate	*p* value
Single	191/204 (94%)	0.1912	27/191 (14%)	0.1557	29/31 (94%)	0.4776	7/29 (24%)	0.7982
Multiple electrode with Switch	19/20 (95%)		3/19 (16%)		6/6 (100%)		3/6 (50%)	

A total of 48 inconspicuous tumors in the HCC group and 6 in the metastatic group were treated with RVS-guided RFA using either single or multiple electrodes with switch. In the HCC group, all (6/6) tumors with anatomical landmark had complete response with 17% (1/6) local recurrence rate. Multiple electrodes with switch were not used in any of the tumors. Seventy-eight percent (31/40) of tumors without landmark achieved a complete response, while all (2/2) of the tumors treated with multiple electrodes had a complete response. The local recurrence rate for tumors with single electrode was at 3% (1/31), while there was none in the tumors treated with multiple electrodes.

In the metastatic group, 50% (1/2) of tumors with anatomical landmark and 75% (3/4) of tumors without landmark achieved a complete response when single electrode was used. No local recurrence was noted, and no tumor in this group was treated using multiple electrodes. Statistical analysis was not done due to low sample size in the multiple electrodes with switch (Table 5).

**Table 5:** Complete response and Local recurrence rate in inconspicuous tumors with use of single and multiple electrodes with switch

Electrode type	HCC *N* = 48				Liver Metastasis *N* = 6			
	Landmark *N* = 6		Without landmark *N* = 42		Landmark N = 2		Without landmark *N* = 4	
	Complete response	Local recurrence rate	Complete response	Local recurrence Rate	Complete response	Local recurrence rate	Complete response	Local recurrence rate
Single	6/6 (100%)	1/6 (17%)	31/40 (78%)	1/31 (3%)	1/ 2 (50%)	0	3/ 4 (75%)	0
Multiple electrodes with Switch	None	NA	2/2 (100%)	0	None	NA	None	NA

Table 6 indicates that RVS-assisted RFA resulted to a high rate of complete response in small to intermediate size tumors. The local recurrence rate showed an upward trend as the tumor size increased however these results were not statistically significant. In spite of this, the use of RVS remains a substantial tool in achieving complete ablation.

**Table 6:** Complete response and local recurrence rate by HCC size

Size	Total N = 272	Complete response	p value	Local recurrence rate	*p* value
< 2 cm	125	116/125 (93%)	0.8374	10/116 (9%)	0.1855
2 – 2.9 cm	93	88/93 (95%)		13/88 (15%)	
3 – 4.9 cm	54	50/54 (93%)		9/50 (18%)	

**Fig. 6:**
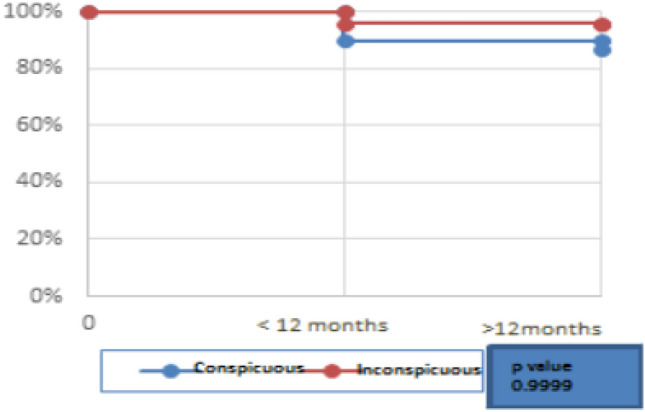
Cumulative local recurrence in Conspicuous and Inconspicuous HCC

The cumulative LR for conspicuous HCC was 10% (23/224) and 13% (23 + 7/224) at less than 12 months and more than 12 months, respectively. The cumulative LR in inconspicuous HCC was 4% (2/48) in less than 12 months, and there was no recurrence incurred after 12 months. The median follow-up was 11 months in both groups. However, the results were not statistically significant (p 0.9999) (Fig. 6).

The cumulative LR in conspicuous liver metastasis was 24% (9/37) and 27% (9 + 1/37) at less than 12 months and after 12 months, respectively. There was no local recurrence in the inconspicuous tumor group. The median follow-up was 6 months. However, they were not statistically significant (p 0.9564).

There were no immediate complications or side effects documented in both patient groups.

## Discussion

Conventional ultrasound of the liver is widely used for numerous interventional procedures. However, ultrasound has limitations in detecting some liver lesions. The results of multivariate analysis by Okamoto 2010 suggested that nodule size, echo pattern, and location are associated with differences in detection. The use of RVS can guide in identification of lesions when B-mode ultrasound cannot adequately characterize these tumors. The benefits include an increased diagnostic confidence, direct comparison of the lesions using different imaging modalities, more precise monitoring of interventional procedures, reduced radiation exposure [21], and increased chances of successful RFA [17]. Real-time virtual sonography have some weakness regarding imaging incompatibility which might be attributed to the fact that the depth of breathholding on CT and US examination varies, and also increases when the distance is greater between the magnetic sensor and the magnetic generator [22].

Our study included 219 patients (see Table 1) with a mean age of 65 years old in the HCC group. A total of 315 tumors were analyzed (see Fig. 1) as outlined by the inclusion and exclusion criteria. They were classified as conspicuous or inconspicuous (see Fig. 2) based on the margin and echogenicity. For inconspicuous tumors, the presence of an anatomical landmark was used in its identification. The presence of liver cirrhosis was a risk factor for the development of HCC as pointed out by the 185 cirrhotic patients who developed HCC (see Fig. 3) wherein Hepatitis B infection (42%) was the leading cause (see Fig. 4). On the other hand, Fig. 5 describes Colorectal cancer (30%) as the etiology of hepatic metastasis.

Wong reported [23] that complete ablation rate without RVS guidance in HCC with distinct margin was 86.9% (166/191) while 64.7% (11/17) in HCC with indistinct margin. Moreover, Lee [15] cited that the use of RVS in inconspicuous HCC achieved complete ablation rate of 87.5% (7/8). The study of Song et al. [16] revealed that 31.7% (26/82) of HCC not visible under image fusion were ablated under the guidance of fusion imaging in which the technique was based on peritumoral anatomic landmarks.

The utilization of landmarks was useful in the precise identification and ablation of inconspicuous liver tumors [16].

Moreover, the success rate of single ablation with US and CT/MRI fusion imaging in inconspicuous tumors (HCC, liver metastasis) was 83.8% [13] – 90.2% [14]. Virtual CT sonography was expected to be useful in patients with hepatic metastasis. The borders of metastatic nodules in the liver frequently are not clear on B-mode sonography due to the lack of a tumor capsule and because of cellular infiltration of metastatic lesions [12]. Complete ablation can be achieved in 90% of metastases with a diameter of ≤ 3 cm [6]. In the present study, the complete ablation rate in liver metastasis was 91% comparable to the HCC group (*p* = 0.1462) (see Table 1).

By comparison, this study showed an increase in the complete response rate after first session of RVS-assisted RFA in conspicuous tumor group at 94%. The complete response rate of RVS-assisted RFA in inconspicuous tumor group was 80% (78% without landmark and 88% with landmark) comparable to the data of Xu and Mauri. The importance of RVS in conspicuous tumors gives added information for the accurate and safe electrode position and its relationship to nearby vital structures. Combining both RVS and use of anatomical landmark in inconspicuous tumors achieved a higher ablation rate (88%). The positional relationship of tumor to these landmarks is valuable for identifying lesions in which conventional ultrasonography cannot detect (see Tables 2 and 3).

In a study by Kitada [11], the local recurrence rate of the RVS-guided RFA group with 24 obscure HCC was similar to that of the conventional RFA group with 39 clear HCC (8.3% vs 7.7%). A larger tumor size of (> 2 or 3 cm) is a risk factor for early local tumor recurrence. The local recurrence rates of small HCCs after RFA were 1.3–12% at 1 year and 1.7–24% at 2 years [24]. The local recurrence rate tends to be low in HCC patients who were proven to have adequate ablation margin after RFA: namely, not only disappearance of vascular enhancement of main tumor, but also an adequate ablation margin [20].

The cumulative local recurrence rates in liver metastasis were 30% at 6 months and 37% at 12 months [6].

Single and multiple electrodes with switch were used in our patients. As shown in Table 4 for the conspicuous tumor group, there was no difference in achieving complete response to ablation and local recurrence rate. However, there was no statistical analysis done in the inconspicuous tumors using either electrodes due to small sample size (see Table 5). Complete response and local recurrence rates in HCC with varied sizes were also statistically analyzed as shown in Table 6 with comparable outcome.

**Fig. 7:**
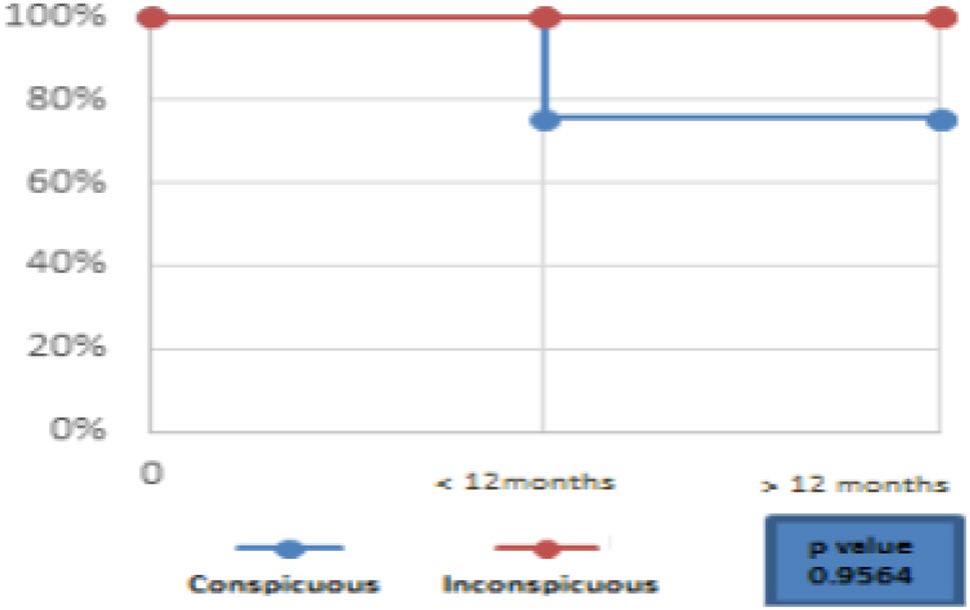
Cumulative local recurrence in Conspicuous and Inconspicuous Liver Metastasis

In contrast, the cumulative LR in the conspicuous HCC group was 10% and 13% at 1 and 2 years, respectively, while cumulative local recurrence in the inconspicuous HCC group was at 4% at 1 and 2 years, respectively. In the liver metastasis group, the cumulative local recurrence in the conspicuous tumor was 24% and 27% at 1 and 2 years, respectively, while no local recurrence was seen in the inconspicuous liver metastasis group. The results show a lower cumulative LR as compared to aforementioned data which could be attributed to the use of RVS (see Figs. 6 and 7).

Logistic regression analysis was done to determine the likelihood for local recurrence in HCC. At 90% level of significance, the size of the tumor was a significant predictor of local recurrence. A one centimeter increase in the size of the tumor can increase the likelihood of local recurrence by 47.9% (*p* = 0.033) (see Table 7).

**Table 7:** Logistic Regression on Likelihood of Local Recurrence for HCC

	B	S.E	Wald	df	*P* value	Exp(B)
Size (cm)	.391	.183	4.564	1	.033	1.479
Constant	− 2.880	.485	35.240	1	.000	.051

By comparison, the 27 tumors with non-complete response during the first RFA showed that most of them were located in high-risk areas (near vessels, subcapsular); however, logistic regression analysis revealed that they were not statistically significant (*p* = 0.21563). This result emphasizes the use of RVS in achieving an increased ablation rate even in high-risk areas both in conspicuous and inconspicuous tumors of variable sizes (see Table 8).

**Table 8:** Logistic regression on likelihood of non-complete response

	B	SE	Wald	Df	*p* value	Exp (B)
Constant	− 1.961	0.603	− 3.252	1	0.001	14%
High-risk location	− 0.5222	0.4217	− 1.238	1	0.21563	59%
Size	− 0.1757	0.2305	− 0.762	1	0.44586	84%

## Conclusion

Real-time virtual sonography achieved a comparable complete ablation rate after the first session of RFA in the HCCA group (92%) and metastatic tumor group (91%). The use of RVS in conspicuous tumors resulted in a higher primary technique effectiveness rate at 94% in contrast to other published data. The relevance of RVS in the treatment of inconspicuous tumors was proportionate to the outcomes of other studies at 80%. Combining RVS in tumors with anatomical landmarks, it further enhanced the detection and ablation of inconspicuous tumors (88%). However, a larger sample size may be needed to validate this claim. Furthermore, RVS-assisted RFA treatment of conspicuous and inconspicuous tumors led to a lower cumulative local recurrence rate.
